# Molecular Insights into HPV-Driven Cervical Cancer: Oncoproteins, Immune Evasion, and Epigenetic Modifications

**DOI:** 10.3390/microorganisms13051000

**Published:** 2025-04-27

**Authors:** Luciana Alexandra Pavelescu, Nicoleta Larisa Mititelu-Zafiu, Dana Elena Mindru, Radu Vladareanu, Antoanela Curici

**Affiliations:** 1 Department of Cellular and Molecular Biology and Histology, “Carol Davila” University of Medicine and Pharmacy, 050474 Bucharest, Romania; luciana.pavelescu@umfcd.ro; 2 Synevo Romania, 021408 Bucharest, Romania; nicoleta.mititelu@synevo.ro; 3 Department of Pediatrics, Faculty of Medicine, “Grigore T. Popa” University of Medicine and Pharmacy, 700115 Iasi, Romania; mindru.dana@umfiasi.ro; 4 Department of Obstetrics-Gynecology and Neonatology, Elias Emergency Hospital Bucharest, 011461 Bucharest, Romania; 5 Obstetrics and Gynecology, University of Medicine and Pharmacy Carol Davila, 050474 Bucharest, Romania

**Keywords:** HPV, miRNA, exosomes, cervical cancer, early proteins, DNA methylation

## Abstract

Cervical cancer ranks third in mortality and fourth in incidence among women worldwide as one of the leading causes of death from cancer in females. The main reason behind cervical carcinogenesis is long-term infection with high-risk human papillomavirus (HPV) genotypes, particularly HPV16 and HPV18. This review investigates HPV distribution across the world, along with cervical cancer molecular development mechanisms and current treatment strategies. Epidemiological data show that disease patterns vary significantly between different geographic regions because underdeveloped nations bear a higher disease burden. The molecular mechanisms of oncogenes E6 and E7 disrupt tumor suppressor pathways, while epigenetic modifications through DNA methylation and miRNA dysregulation promote malignant cell transformation. The reduction in HPV infection through prophylactic vaccination has shown promise, yet barriers related to accessibility and coverage still exist. The therapeutic technologies of gene expression inhibitors together with immunotherapies and epigenetic targeting agents show promise but require optimization to achieve specific targeting while minimizing off-target effects. A combined approach that integrates HPV vaccination with early diagnosis and molecular-specific therapies represents the most effective method to manage cervical cancer impact. The future care of patients will require increased translational research along with better immunization programs to drive prevention and therapeutic outcomes.

## 1. Introduction

### 1.1. Prevalence and the Impact of HPV on Cervical Cancer

Human papillomavirus (HPV) remains a critical global health concern due to its well-established etiological role in CC, which ranks as the fourth most frequently diagnosed cancer and the third leading cause of cancer-related mortality among women worldwide. According to GLOBOCAN 2022 data, an estimated 662,000 new cases of CC and approximately 350,000 deaths occurred globally that year. The burden of CC is disproportionately high in low- and middle-income countries (LMICs), which account for nearly 88% of new cases and 90% of CC-related deaths, primarily due to inadequate access to HPV vaccination, screening programs, and timely medical interventions. Persistent infection with high-risk HPV genotypes, particularly HPV 16 and 18, is responsible for nearly 70% of all CC cases worldwide. Given the substantial global burden of HPV-associated malignancies, a deeper understanding of its molecular pathogenesis, immune evasion strategies, and epigenetic alterations are essential for advancing prevention, early detection, and management strategies [1,2].

The epidemiology of human papillomavirus (HPV) infection demonstrates significant geographical variability, with notable differences between developed and emerging regions. In Western Europe, the prevalence of HPV infection is estimated to be 3.7%, with most European nations exhibiting low prevalence rates (below 30%), albeit there being certain exceptions. In contrast, HPV prevalence is markedly higher in emerging regions, reaching 42.2% compared to 22.6% in developed regions. Furthermore, independent of socioeconomic development, certain areas exhibit persistently elevated HPV prevalence, such as Eastern Europe (21.4%). Conversely, North Africa (9.2%) and Western Asia (2.2%) demonstrate lower infection rates [3,4,5,6]. In sub-Saharan Africa, HPV prevalence is estimated to be 24.4% [7], while in Ethiopia, approximately 17.3% of the population is infected with HPV, with high-risk strains comprising 15.8%. Notably, 54% of these CC cases occur in Asia, underscoring the substantial public health challenge posed by CC in the region [8,9,10,11].

Persistent high-risk HPV infection is responsible for nearly all cases of CC, with approximately 95% of cervical malignancies occurring in women who do not receive treatment for chronic HPV infection. Among immunocompromised individuals, particularly those with untreated HIV, disease progression may be significantly accelerated, reducing the transition time from persistent HPV infection to invasive cancer from the usual 15–20 years to as little as 5–10 years.

The global burden of high-risk HPV infection is subject to substantial epidemiological variation, influenced by factors such as geography, socioeconomic status, cultural practices, HPV genotype distribution, age, and host immune competence. Overall, high-risk HPV accounts for approximately 11.7% of infections worldwide, with prevalence exceeding 35.4% in some developing regions [12,13]. A more comprehensive understanding of the molecular interactions between HPV and host immune mechanisms is critical for elucidating the oncogenic potential of HPV and improving strategies for CC prevention and management.

A study encompassing diverse regions such as Europe, the Americas, and Africa demonstrated that women aged 45 years and older exhibit the second highest prevalence of HPV infection, with 32% of this demographic being infected with high-risk HPV types, namely HPV16 or HPV18, or a combination of both [14].

Worldwide, approximately 291 million women are estimated to harbor HPV DNA. A wealth of evidence supports the association between HPV and various risk factors, including multiple sexual partners [15], high parity [16], early pregnancy [17], concurrent sexually transmitted infections [18], the use of hormonal contraception [19], and tobacco smoking [20,21].

**Figure 1:**
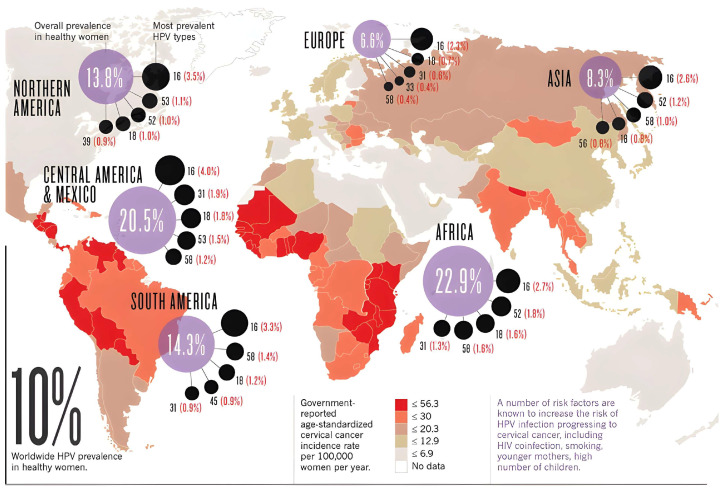
World map with the prevalence of the predominant high-risk HPV subtypes by continent or region in healthy women (Adapted from: https://www.cancer.gov/about-nci/organization/cgh/blog/2018/world-report, accessed on 1 February 2025).

Globally, HPV is responsible for approximately 31% of all cancer cases, and it contributes to 13% of all infection-related cancers [22]. HPV types 16, 18, 26, 31, 33, 35, 39, 51, 52, 58, 59, 66, 68, 73, and 82 are considered carcinogenic to humans (Figure 1) [23]. In India, the substantial burden of cancer is attributed to HPV, with approximately 24% of all cancer cases in 2020 being linked to HPV infection, accounting for 7% of the global cancer burden [24].

### 1.2. HPV Classification

More than 40 HPV genotypes exhibit tropism for the anogenital epithelium [25,26]. According to the IARC Monograph 100B [2012] and subsequent updates, these are classified as follows:

**Group 1** (Carcinogenic): HPV 16, 18, 31, 33, 35, 39, 45, 51, 52, 56, 58, 59, and 68 [27,28].
Mechanism: E6/E7-mediated p53/Rb degradation, genomic instability.
**Group 2A** (Probably carcinogenic): HPV 68 (previously listed here; now reclassified to Group 1), with no other genital HPV types currently assigned to Group 2A by IARC [27,29]. Some studies propose HPV 26, 53, or 66 for Group 2A, but IARC retains them in Group 2B due to insufficient evidence [26,28].
**Group 2B** (Possibly carcinogenic): HPV 26, 53, 66, 67, 70, 73, and 82 [28,30].
**Group 3** (Not classifiable/Low risk): HPV 6, 11, 40, 42, 43, 44, 54, 61, 72, 81, and 89 [28,30].

Current IARC Monographs list no genital HPV types as Group 2A. Non-genital types (e.g., HPV 5, 8 for skin cancer) may be included here.

However, standardized classification remains elusive for many low-prevalence HPV genotypes due to insufficient oncogenicity data [25,31]. Globally, HPV-16 and HPV-18 account for approximately 70% of CC cases, with molecular studies confirming their dominant etiological role through E6/E7 oncoprotein-mediated carcinogenesis [25,32].

Notably, meta-analyses demonstrate modest but consistent geographic variation in HPV-16/18 attribution, accounting for 72–77% of cervical cancers in high-income countries compared to 65–72% in low- and middle-income countries [27,33]. This disparity underscores the need for refined classification criteria incorporating the following: (1) population-specific genotype distributions, (2) molecular markers of oncogenic progression (e.g., viral integration status, E6/E7 expression levels), and (3) longitudinal persistence data [26]. Such multidimensional stratification would improve risk prediction beyond the current dichotomous (high/low-risk) systems.

Based on their association with cervical cancer and precursor lesions, HPVs can also be grouped into high-risk and low-risk HPV types. Low-risk HPV types include types 6, 11, 42, 43, and 44. High-risk HPV types include types 16, 18, 31, 33, 34, 35, 39, 45, 51, 52, 56, 58, 59, 66, 68, and 70. Included in the high-risk group are some HPV types that are less frequently found in cancers, but are often found in squamous intraepithelial lesions [SILs]. Some authors refer to these HPV types as intermediate risk. Low-risk subtypes are also occasionally found in cervical carcinomas [34]. The current diagnostic paradigms for cervical HPV infection and carcinogenesis rely on viral DNA detection in cervical scrapes or biopsy specimens via PCR-based assays (e.g., Cobas^®^ HPV Test, Linear Array) [31].

The molecular evolution of HPVs reveals an intricate diversification shaped by genomic drift, host adaptation, and lineage-specific selective pressures. Early comparative phylogenetic analyses across 48 HPV types and 28 subtypes highlighted profound evolutionary divergence among HPV clades, with lineage-specific genetic signatures being evident even within closely related types. More recent genome-wide analyses have reinforced this complexity, using molecular evolutionary genetics frameworks to elucidate subtype-specific microRNA repertoires and revealing that viral genomic regions involved in oncogenesis exhibit accelerated evolution relative to structural genes [35].

Phylogenetic reconstructions focusing on HPV16-related types, including HPV31, HPV33, and HPV58, have demonstrated that subtype diversification often parallels geographic and ethnic population structures, suggesting the co-migration of viral lineages with human hosts [36]. Analyses of Alpha papillomavirus 7 species (HPV18, HPV45, and HPV59, among others) revealed an ancient phylogenetic root in Africa, followed by a global radiation associated with human dispersal events [36].

Notably, phylogenetic trees constructed for HPV18 isolates revealed distinct African and European lineages, reflecting both ancient divergence and recent epidemiological dynamics [37]. Comparative studies on variants of HPV types 18, 45, and 97 further illustrate that intratype genomic variations are not stochastic, but reflect distinct evolutionary histories shaped by founder effects and host–virus interactions [36].

Thus, phylogenetic frameworks provide a robust lens for interpreting the molecular evolution of HPV, revealing that HPV subtypes and variants represent natural, ancient viral taxa with deep evolutionary roots, intertwined with human demographic expansions [38].

### 1.3. Significance of HPV as a Public Health Concern, Particularly Its Association with CC

Human papillomavirus [HPV] is established as the necessary cause of CC, with persistent infection implicated in 99.7% of high-grade precarcinomas. While approximately 70% of sexually active women acquire genital HPV infections, 90% undergo spontaneous clearance within 1–2 years due to robust cell-mediated immunity, posing no long-term risk [39,40].

Many nations now adopt HPV genotyping as the primary screening modality, superseding conventional cytology (Papanicolaou testing) due to superior sensitivity for detecting precancerous lesions [10]. However, the low specificity (~85%) of HPV DNA testing can generate unnecessary colposcopies [10].

To mitigate overdiagnosis, secondary triage methods are imperative, including the following:

- Cytology co-testing (e.g., Pap smear for HPV-positive cases);
- p16/Ki-67 dual staining (improves specificity for CIN2+);
- Methylation markers (e.g., FAM19A4/miR124-2) for progressive lesions.

### 1.4. Pathogenesis of HPV

The transition from a normal epithelium to invasive cervical carcinoma is a long and multi-step process, involving viral integration, the aberrant regulation of the host cell cycle, immune avoidance, and metabolic rewiring.

HPV is a small, spherical, non-enveloped, double-stranded DNA virus that belongs to the Papaviridae family. Its diameter ranges from around 50 to 55 nanometers. A total of 72 capsomers are contained within this structure, which is an icosahedral capsid, as stated by Baker et al. [41,42].

According to Stanley et al., the genome of HPV is circular and is separated into three functional parts [43]. These segments are the non-coding long control region [LCR], the early (E) region, and the late (L) region. Favre and Torrisi et al. found that the late region is responsible for the production of the structural proteins L1 and L2 [41,44]. It is the LCR that controls the expression of viral genes and the replication of DNA. The early region is responsible for the encoding of proteins (E1, E2, E4, E5, E6, and E7) that play a role in the replication of viruses and the occurrence of carcinogenic events [45,46,47,48].

## 2. Early Proteins and Viral Integration Process

### 2.1. E1 and E2 Proteins: Initiation of HPV Replication and Viral Integration

Viral receptors on host cells let infection start in the basal layer of the squamous epithelium. The viral genome is originally housed in the nucleus in an episomal form, which allows the virus to replicate inside the host cell like a plasmid. The strict control of infection across the early phases is attributed to the E1 and E2 proteins [49,50,51].

E1 protein, which is essential for the beginning of viral DNA replication, shares a significant degree of similarity across different HPV types. It functions as a helicase and is essential for viral replication, since it unwinds the DNA of the virus. Upon recruitment to the origin of replication, E1 initiates the formation of a complex with E2, the principal regulatory protein of HPV replication [52,53,54,55,56,57,58,59,60].

E2 is a multifunctional protein, as it is needed both for the transcriptional regulation of the viral genes, as well as for the integrity of the viral genome during replication. By binding to the E2 binding sites (E2BS), E2 recruits E1 and forms the E1–E2 complex. It is possible to start the replication process once this complex has finished unwinding the DNA. During the integration of the viral genome into the host’s genome, the E2 gene is often disrupted, leading to the loss of its regulatory functions and the unregulated activation of the E6 and E7 oncoproteins [52,55,56,57,58,59,60,61,62,63,64,65].

The disruption of E2 in the viral life cycle is a signature of HPV integration and cancer. In fact, 83% of HPV-positive cases have the viral genome integrated into the host itself. The biology of this process has been studied in depth. The HPV genome undergoes partial replication via E1 and E2, which can trigger DNA damage response (DDR) pathways and cause genome damage [66,67]. Upon the uncontrolled nuclear expression of E1, DNA damage leads to the activation of kinases including the recruitment of the ATR and ATM kinases, which promote double-stranded DNA breaks (DSBs) and enable the integration of HPV. In addition, E2 also targets nucleases to its binding sites in the long control region (LCR), producing DSBs which may account for the shortened HPV genomes found in CC [68,69].

A different theory involves the hyperactivity of apolipoprotein B mRNA-editing enzyme catalytic polypeptides (APOBEC), which can cause hypermutation, disrupt E2, and facilitate the integration of HPV DNA into the host genome [69,70,71]. More studies indicate that E1 causes genomic instability locally because of DNA overamplification, and that E2 mediates E6-induced apoptosis; these findings suggest strategies that HPV uses to hijack host cellular events and facilitate its life cycle.

The degradation of E2 leads to the overexpression of E6 and E7, which obliterates tumor suppressor proteins like p53 and retinoblastoma (Rb) [72,73]. The disruption of the cell cycle and the inactivation of apoptotic mechanisms lead to a cancerous transformation of these infected cells [73,74].

### 2.2. E4 Protein: Role in Cytopathic Effects and Viral Release

The E4 protein is a product of the alternative splicing of the E2 gene and is an important player in late viral infections. E4, which is mainly expressed in the upper layers of the epithelium, plays a role in the amplification of the viral genome, as well as in the disintegration of host cellular structures. Interaction with host cell keratin filaments is one of the major functions of E4. E4 is tethered to keratin via an N-terminal leucine cluster motif, which also destabilizes the keratin cytoskeleton and enables the budding of new virions from the infected cells [75,76,77,78,79,80,81,82].

In addition, E4 is involved in cytoplasmic granule formation, which is implicated in the cytopathic effects of HPV-infected cells. In the most straightforward terms, this impact varies from generating cell hyperplasia to having an impact on an intracellular and extracellular level, which is known to be involved in virus pathogenicity. Moreover, E4 is subject to various forms of post-translational modification like phosphorylation, which is known to increase its stability and ability to cohesively bind keratin. The S phase-dependent phosphorylation of E4 by cyclin-dependent kinases (CDKs) in turn helps to modulate the host cell cytoskeletal network to facilitate the release of nascent virions while also playing a role in controlling viral replication itself [51,69,83,84].

### 2.3. E5 Protein: Modulation of Immune Response and Cell Growth

The E5 is a small hydrophobic transmembrane viroporin involved in viral replication and modulating signaling pathways. One of its major mechanisms is the activation of the epidermal growth factor receptor (EGFR), resulting in downstream signaling through the PI3K-AKT and MAPK pathways, leading to cell growth and survival. This dysregulation causes the uncontrolled proliferation of infected cells. E5 is also important in immune evasion, helping HPV to evade host defense mechanisms. It inhibits interferon (IFN) signaling by binding to a stimulator of interferon genes (STING), which is associated with the decreased activation of the JAK/STAT pathway [51,84,85,86,87].

E5 also downregulates the CDK inhibitors p27^Kip−1^ and p21^Waf−1^, enabling infected cells to avoid cell cycle arrest and divide further. Additionally, it inhibits keratinocyte differentiation by downregulating keratinocyte growth factor receptor (KGFR), and at the same time, increases EGFR activity, leading to delayed differentiation and the maintenance of keratinocyte proliferation [88,89,90].

E5 inhibits molecules of the major histocompatibility complex class I (MHC I) to evade immune system identification, hence diminishing the functionality of T cells and natural killer (NK) cells. To evade detection by the immune system, HPV inhibits the synthesis of viral antigens on the surfaces of infected cells [91].

In addition, E5 also has been shown to upregulate the expression of VEGF, thereby stimulating angiogenesis, which supplies the growing tumor.

### 2.4. E6 Oncoprotein: Regulator of Tumor Suppression and DNA Repair

The E6 and E7 oncoproteins are the most largely studied HPV proteins associated with cancer. These proteins serve as the main drivers of HPV carcinogenesis.

E6 is a potent oncoprotein, promoting carcinogenesis via the suppression pathways, primarily through the degradation of p53. E6 binding to E6AP (an E3 ubiquitin ligase) causes p53 degradation, eliminating cell cycle inhibition at the G1 checkpoint and allowing infected cells to replicate uncontrollably [92]. It can also induce an upregulation of OCT-4 that not only inhibits the activity of p53 but also promotes stem cell-like characteristics in infected cells [93,94,95].

In addition to inhibiting apoptosis, E6 is also involved in manipulating DNA damage repair mechanisms, promoting increased genomic instability. It perturbs essential proteins involved in DNA damage responses, including CHEK2, CLK2/3, ERCC3, MNAT1, PER1, RMI1, RPA1, UVSSA, and XRCC6, which facilitates viral amplification but damages the host genome. E6 enhances telomerase activity by degrading NFX1-91, a known telomerase inhibitor, subsequently activating hTERT. This process allows cells to bypass senescence and achieve immortality [92,96].

E6 further affects key signaling cascades involved in cell survival and proliferation, including PI3K/AKT and Wnt/β-catenin. E6 promotes the degradation of tumor suppressor proteins (hDLG, hSCRIB, MAGI-1, -2, -3, and NHERF-1) via its PDZ-binding motif, resulting in the uncontrolled activation of pro-survival and mitogenic cascades. Together, these molecular perturbations drive cellular transformation and tumor progression [61,97,98,99,100,101,102].

### 2.5. E7 Oncoprotein: A Driver of Cell Cycle and Genome Instability

E7 acts mainly by inactivating the tumor suppressor protein retinoblastoma (pRB), allowing E2F transcription factors to trigger the S-phase of the cell cycle. E7 does so by recruiting the cullin 2 (CUL2) ubiquitin ligase to degrade pRB. This enables HPV-infected cells to multiply uncontrollably [100,103,104].

It also upregulates the expression of the eIF4E translation factor, which promotes the protein synthesis needed for viral replication. Moreover, E7 directly regulates the G2/M checkpoint via histone H1 kinase activity modulation, creating an optimal environment for viral genome replication on the cell cycle front, while promoting cellular genome instability [99,100,102].

In addition, E7 takes advantage of the DNA damage response that is begun by RN1698 E3 ubiquitin ligase in order to assist the virus in replicating (this ultimately poses a danger to the integrity of the host genome). Furthermore, E7 is responsible for activating the PI3K/AKT/SGK signaling cascade, which is responsible for facilitating cell migration, survival, and the transition from epithelial to mesenchymal cells. It is evident from this that E7 plays a significant part in the pathway that leads to the formation of tumors that are connected to HPV [105,106,107,108].

**Figure 2:**
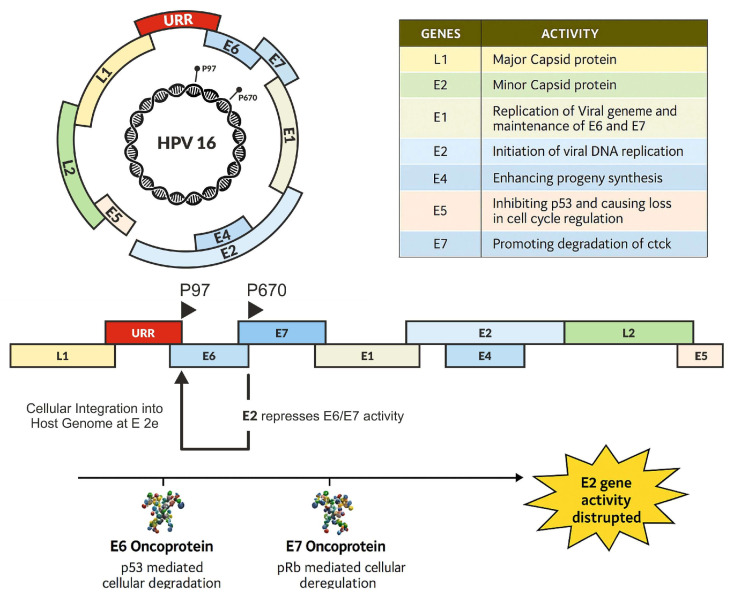
The mechanisms of oncoproteins in carcinogenesis.

Aside from their role in the cell cycle and apoptosis, the E6 and E7 proteins contribute to other hallmarks of cancer, including genomic instability, immune evasion, and cell invasion. E6 leads to genomic instability through mutagenesis and the inhibition of DNA repair, and E7 promotes invasion by stimulating extracellular matrix degradation. These proteins act together to drive cells infected with HPV down the path from normal cells to malignant transformation, and ultimately to HPV-associated cancers (Figure 2) [51,109,110].

### 2.6. Late Proteins—L1 and L2

The structural proteins of the viral capsid are encoded by the late (L) region of the genome, and consist of two components: L1, the major capsid protein, and L2, the minor capsid protein. [90,111].

The principal capsid protein, L1, is structured in 72 pentameric subunits termed capsomeres, each consisting of five L1 monomers, culminating in a total of 360 L1 molecules per mature virion. The minor capsid protein, L2, exists in a reduced quantity, with roughly one molecule per capsomere—amounting to approximately 72 L2 molecules per capsid [112]. L1, a protein of approximately 55 kDa, is crucial for capsid assembly, serves as the principal structural element of the virion, and enables viral attachment by binding with heparan sulfate proteoglycan receptors on keratinocyte surfaces. Following receptor activation, the viral capsid undergoes conformational changes, which are essential for the subsequent entry processes. Proteolytic cleavage of L1 and L2 by host cell enzymes transpires before or during cellular entrance, facilitating the effective uncoating and internalization of the viral genome [111].

The regulation of viral replication and transcription is mediated by a non-coding segment known as the Long Control Region (LCR), which makes up about 10% of the human papillomavirus (HPV) genome. It is found between the open reading frames L2 and E6. The LCR contains several conserved regulatory and tissue-specific elements arranged into three separate domains: the distal domain, the keratinocyte-specific (KE) domain, and the proximal domain [113]. Many transcription factor binding sites in the KE domain control RNA polymerase II-driven transcription from both early and late promoters, hence enhancing their value. Among others, they comprise binding motifs for transcription factors AP-1, NF-1, TEF-1, OCT-1, YY-1, BRN-3a, NF-IL6, KRF-1, NF-κB, FOXA1, and GATA3 [90]. Three E2 binding sites found in the proximal domain are vital for the control of viral transcription and replication. Binding sites for basal transcriptional machinery abound in this region as well: Sp1, the TATA box, and the early promoter. Moreover, this proximal area contains the origin of replication (ori), necessary for viral DNA replication [90].

### 2.7. HPV Signaling Pathway

Important for the development of cancer, the HPV oncoproteins E5, E6, and E7 modulate several cellular signaling pathways. These pathways show interesting targets for the development of new therapeutic approaches since they are central in malignant transformation. While E6 disturbs several important molecular pathways, including the PI3K/AKT, Wnt, and Notch pathways, the HPV E5 oncoprotein activates the MAPK-ERK signaling pathway [97]. The E7 oncoprotein also tunes the PI3K/AKT axis. Particularly important in malignant transformation, the PI3K/AKT pathway drives cell proliferation, metastases, and drug resistance.

### 2.8. MAPK-ERK Pathway

The E5 oncoprotein has been previously described to activate the MAPK-ERK pathway [97]. Involved in cell proliferation and differentiation, this pathway is critical, as MAPK-ERK activation leads to enhanced expression of oncogenes like c-Fos for cancer cell abiding and metastasis. Moreover, SRC proto-oncogene and phosphorylated ERK1/2 also modulate ERK signal transduction to play a role in tumor progression [51,114].

There are several therapies currently undergoing clinical trials targeting the vascular endothelial growth factor (VEGF) and EGFR for CCs associated with HPV. It has been reported that HPV16 E6 oncoprotein induces hypoxia-inducible factor 1-alpha (HIF-lα) expression, as well as downstream target genes such as VEGF and interleukin-8 (IL-8) through the ERK1/2 signaling pathway [61,102]. Furthermore, high HER3 expression driven by E6/E7 correlates with poor CC prognosis [115]. HER3 mRNA and protein expression are positively correlated in HPV-mediated cervical carcinoma through PI3K signaling [116].

### 2.9. PI3K/AKT, Wnt, and Notch Pathways

The phosphatidylinositol 3-kinase (PI3K)/AKT signaling system is important in promoting cell division, the maintenance of viability, and metabolic adaptation. The E6 oncoprotein mainly modifies the phosphatidylinositol 3-kinase and protein kinase B (PI3K/AKT) pathway, which then leads to cell proliferation and drug resistance [98,117]. The E6 protein can also inactivate tumor suppressor proteins such as p53 or PTEN [118,119], which leads to the dysregulation of the cell cycle. Abnormal activation suppresses apoptosis, accelerates cellular proliferation, and aids processes like the epithelial–mesenchymal transition (EMT), thus fostering malignant transformation.

The HPV E6 oncoprotein activates the Wnt/β-catenin signaling pathway, which is essential for epithelial cell immortalization, cell proliferation, and tissue homeostasis. Under typical circumstances, β-catenin levels are rigorously regulated by proteasomal degradation; however, E6-induced activation results in the stabilization and nuclear accumulation of β-catenin, hence facilitating the transcription of oncogenic target genes such as c-Myc and cyclin D1. Aberrant activation of the Wnt/β-catenin pathway has been associated with the onset and persistence of HPV-induced malignancies, leading to the disruption of the normal epithelial architecture and the development of malignant phenotypes. Furthermore, sustained Wnt/β-catenin signaling enhances the stem-like characteristics of cancer cells, contributing to tumor growth and resistance to therapy. The Wnt/β-catenin pathway, due to its pivotal involvement in cervical carcinogenesis, constitutes a prospective therapeutic target for cervical cancer treatment [120].

The Notch signaling pathway, recognized for its oncogenic role in leukemia and other cancers, paradoxically has tumor-suppressive activity in hr-HPV-induced cervical carcinogenesis [92,107,121]. E6 has an inhibitory role in the Notch signaling pathway, which promotes tumor progression via the loss of p53-mediated cellular control. Notch and p53 participate in a reciprocal regulatory loop, whereby Notch activation stabilizes p53, and its dysregulation fosters cancer. The Notch signaling pathway was shown to cooperate with the PI3K-AKT pathway to promote cellular progression, highlighting the intricate role of Notch signaling in HPV-associated carcinogenesis [51,92,119,121].

The E7 protein inactivates retinoblastoma protein (pRB) and causes uncontrolled cell proliferation and the progression of CC [61]. E7 also inhibits p27 through AKT, which promotes its retention in cytoplasmic domains, leading to an increase in cell cycle progression [51,92,122,123].

### 2.10. STAT3 and EMT Pathways in HPV-Induced Cancer

Signal transducer and activator of transcription 3 (STAT3) is one of the most important molecules involved in epithelial cancer progression. Numerous studies have indicated that the binding of STAT3 to the HPV16 LCR regulates the expression of viral oncogenes, thereby influencing cancer development [92,105,124]. HPV16 E6/E7 oncoproteins can also cause EMT through upregulated fibroblast growth factors (FGF)-2 and FGF-4, thereby promoting invasive cancer growth [125]. E-cadherin is a key epithelial differentiation marker and is known to be suppressed by E6/E7 and ErbB-2 during the epithelial–mesenchymal transition progression, which is further activated by central transcription factors such as SLUG, SNAIL, and TWIST [100,105].

### 2.11. Role of AP-1 and Notch Signaling Pathways in Cervical Carcinogenesis

The transcription factor Activator Protein-1 (AP-1) is essential for the expression of HPV oncogenes such as E6 and E7 and plays a pivotal role in the progression of HPV-associated cervical cancer [125]. Studies have identified two activator protein-1 (AP-1) binding sites within the LCR of HPV18, indicating the regulatory role of the AP-1 in viral oncogene expression. The key component of the AP-1 complex, c-Jun, is known to promote tumorigenesis, cell proliferation, invasion, migration, and angiogenesis. Moreover, c-Jun has been specifically associated with the enhanced expression of E6 and E7. In fact, mutations in AP-1 within the HPV upstream regulatory region (URR) can disrupt E6/E7 gene expression [126], emphasizing the importance of this transcription factor in CC progression. AP-1 alterations, such as the increased expression of c-Fos, facilitate tumorigenesis [69,118].

### 2.12. Strategies of Immune Evasion by HPV-Infected Cells

The ability of HPV to evade the host’s immune response is a key factor in the transition from chronic infection to CC. The occurrence of an HPV infection activates mechanisms of viral immune evasion, leading to a range of diseases, including chronic inflammation and the progression of cancer.

The detection and eradication of cells infected with HPV are frequently attributed to both innate and adaptive immune responses. On the other hand, HPV utilizes various mechanisms to escape immune recognition, impede antiviral defenses, and modify host immune responses, ultimately leading to tumor development and chronic infections.

### 2.13. Evasion of Antigen Presentation and Adaptive Immunity

The presentation of antigens to cytotoxic T lymphocytes (CTLs) via MHC class I molecules is a crucial process in the immunological elimination of HPV-infected cells. MHC class I downregulation by E5 and E7 HPV-derived proteins prevents the activation of antigen-specific CTLs. Moreover, HPV-infected keratinocytes prevent the migration of Langerhans cells by decreasing macrophage inflammatory protein-3α (MIP-3α) expression, leading to a decrease in antigen presentation. E6 further impairs immune recognition by preventing the differentiation of monocytes into dendritic cells [127,128].

### 2.14. Innate Immunity and Pattern Recognition Receptors (PRRs)

Keratinocytes, which are non-professional antigen-presenting cells (APCs) that activate cell-mediated immunity, are the first cells to initiate the innate immune response to an HPV infection. Since the multiplication of HPV in keratinocytes does not result in the lysis of the cells, it is possible for these cells to display viral peptides on MHC class I molecules for the purpose of immune system examination.

Even though keratinocytes often do not synthesize MHC class II molecules, there are a few cytokines that have the potential to trigger their production. One of these cytokines is interferon-gamma (IFN-γ), which plays a significant role in the activation of Th1 cells and has a significant impact on the regulation of immunity [129,130,131]. Among the pattern recognition receptors (PRRs) that are found in keratinocytes are Toll-like receptors (TLRs), which play an essential role in the identification of viral DNA. To be more specific, TLR9 is responsible for recognizing double-stranded HPV DNA and triggering the production of type I interferons (IFN), tumor necrosis factor-alpha [TNF-α], interleukin-18 (IL-18), and a variety of chemokines (CCL2, CCL20, CXCL9) [91,132,133,134,135]. This process ultimately leads to the activation of immune cells such as natural killer (NK), natural killer T cells (NKT), leukocyte cell tumor (LCT), macrophages, and dendritic cells (DCs). Macrophages and dendritic cells are examples of professional antigen-presenting cells. These cells phagocytose and deliver HPV antigens to T cells, which activates CD4+ helper T cells and CD8+ cytotoxic T cells, which in turn drives the generation of antibodies by B cells. PRRs are responsible for the identification of HPV by antigen-presenting cells (APCs). When these cells are activated, they release co-stimulatory chemicals and cytokines, which are essential for the generation of an appropriate immune response as well [136,137].

### 2.15. T Cell Responses and Adaptive Immunity

While innate immunity is critical for the early detection and control of HPV, long-term immunity is attributed to the adaptive immune system. CD4+ T helper (Th) cells and CD8+ cytotoxic T lymphocytes (CTLs) are typically elicited against HPV infections. These T cells recognize and destroy cells with HPV-infected cells [134,137,138].

- Th1 cells, which are CD4-positive T helper cells responsible for beginning cellular immune responses, generally facilitate viral clearance after HPV infection. HPV utilizes many mechanisms to avoid host immune responses, mostly facilitated by the viral oncoproteins E5, E6, and E7. Th2 cells are also involved in this process. Nonetheless, HPV infection induces a change in the differentiation of Th1 and Th2 cells towards a Th2 phenotype. This shift likely contributes to both the persistence of HPV infection and the development of cervical lesions. This modification results in a diminished immune response, perhaps accounting for both instances [137];
- CD8+ Cytotoxic T Cell (CTL): CTLs are important for directly killing HPV-infected cells. The E5 and E7 oncoproteins diminish the production of MHC class I molecules, obstructing antigen presentation and resulting in weak or missing HPV-specific CTL responses, commonly seen in persistent HPV infections [139]. In those who have genital warts or CIN, HPV-specific CD8+ T cells can be correlated with the regression of lesions and clearance of the virus. In cases of persistent infection, however, HPV-specific CTL responses are frequently weak or absent, and therefore contribute to an inability to clear the infection [139];
- Tregs function in immune suppression, and their higher numbers in the tumor microenvironment correlate with poor immune responses, as well as persistent HPV. E7 has been associated with the promotion of Treg growth, wherein Tregs secrete IL-10 and TGF-β, which downregulate effector T cell responses and enhance viral immune tolerance. E5 and E6 concurrently facilitate the recruitment of tumor-associated macrophages (TAMs), which release pro-tumor cytokines (including IL-10, VEGF, and TGF-β) and inhibit anti-tumor immune responses [140,141,142,143,144].

HPV establishes a highly effective immune evasion network by modulating Th1/Th2 differentiation, suppressing CTL activity, expanding Tregs, and recruiting TAMs, primarily through the actions of E5, E6, and E7 oncoproteins, thus enabling persistent infection and the progression of cervical cancer.

### 2.16. Activation and Mechanisms of Cytotoxicity of NK Cells

Natural killer (NK) cells are essential to eliminate HPV-mediated and malignant cells. Their activation is the result of a balance between inhibitory and activating receptors. MHC class I molecules typically bind inhibitory receptors such as KIR-L and NKG2A to downregulate NK cell activity. Indeed, to avoid T cell responses, HPV-infected tumor cells frequently downregulate MHC-I expression through the actions of viral oncoproteins like E5 and E7, thus releasing inhibitory signals and stimulating NK cells. NK cells can recognize stress or damage-associated receptors on CC cells, such as MICA/MICB and ULBPs [1,2,3,4,5,6], which engage with NKG2D, CD95 (which binds CD95L), and B7-H6 (which connects with NKp30). Fc receptors (CD16) facilitate antibody-dependent cytotoxicity (ADCC), while tumor-associated CD112 and CD155 interact with DNAM-1 to enhance NK cytotoxicity inside the tumor microenvironment. E6 and E7 not only downregulate MHC-I, but also modulate the expression of NKG2D ligands to evade NK cell surveillance, creating a complex interplay between viral immune evasion strategies and innate immune activation. [145,146,147,148,149,150].

### 2.17. Humoral Immunity and Antibody Responses

Both B cells and antibodies are responsible for the production of humoral immunity, which is the secondary component of the adaptive immune response to HPV. The virus is responsible for the production of antibodies that target capsid proteins (L1 and L2). By rendering the virus ineffective, these antibodies will prevent further infection from occurring. As a result of the fact that HPV is most prevalent in epithelial cell layers, which are areas where immune monitoring is restricted, antibody responses to HPV sometimes prove to be inadequate for eradicating preexisting infections [151,152].

### 2.18. Immunosuppressive Microenvironment and Host Genetic Factors

The host’s genetic characteristics influence both the sensitivity to chronic HPV infection and the likelihood of progression to cervical intraepithelial neoplasia (CIN), alongside viral immune evasion strategies.

Variants in HLA class I and II genes are associated with variations in immune responses to HPV. Increasing research on genetic susceptibility to cervical cancer has focused on the function of HLA II alleles in modulating immune responses to persistent hrHPV infection. Whereas Class II HLA molecules present HPV-derived peptides to CD4+ helper T cells, Class I HLA molecules present them to CD8+ cytotoxic T lymphocytes [109,141,153,154].

Many alleles have been found as either protective markers or risk factors for cervical neoplasia. Among those regularly linked to increased cervical cancer risk are HLA-DRB1*15 and DQB1*0602, as well as DRB1*0401, DRB1*0403, DQB1*0302, D QB1*0402, DQA101:02 and DQA102:01, and D QB1*0603. These alleles are believed to either promote a suboptimal immune response or fail to sufficiently present HPV antigens to CD4+ T helper cells, thus allowing viral persistence and the progression of cancer.

DQA105:01 was associated with significantly reduced odds of prevalent hrHPV infection, yet paradoxically, its presence was linked to an increased risk of persistent infection—a critical determinant of cervical cancer risk. Several haplotypes containing DQA105:01 also showed strong associations with persistent hrHPV infection. This suggests the dualistic role of this allele in modulating both initial immune resistance and long-term immune evasion by the virus, potentially through mechanisms that impair antigen presentation or cytotoxic T cell activation.

Some alleles, on the other hand, show protective actions. Reduced risk of cervical cancer has been linked to DRB1*1301, DQB1*0603, DQB1*0501, and DQA1*0301. These protective alleles probably improve antigen presentation’s efficiency, therefore encouraging viral clearance and a stronger anti-HPV immune response. Fascinatingly, DQB1*0603 has been shown in several studies to be both a risk and protective allele, suggesting possible variation in its functional relevance depending on environmental interactions or genetic background.

Several single-nucleotide polymorphisms (SNPs) in immune response genes, cell cycle regulators, and hormone pathways have been identified as important modulators.

The -1082 A > G polymorphism in the IL-10 gene promoter is associated with reduced immune effectiveness against HPV, thereby increasing cancer susceptibility [155].

Furthermore, a reduced ability to recognize and clear HPV infections has been linked to SNPs in TLR4 (Asp299Gly) and TLR9 (-1486T/C), which promote viral persistence [156].

Among the most investigated polymorphisms are those in the TP53 tumor suppressor gene, especially the Arg72Pro SNP. This variant influences p53’s sensitivity to degradation, driven by HPV E6. The HPV E6 oncoprotein breaks down the Arginine variant (Arg72) more effectively than the Proline variant, thus increasing the risk of cervical cancer in Arg72 carriers [157].

Though not usually in the general population, somatic alterations in p16INK4a, a cyclin-dependent kinase inhibitor, occur in cervical precancerous lesions. Loss of the p16INK4a function causes uncontrolled cell cycle progression, thus promoting malignant transformation.

Variations in the estrogen receptor α gene (ESR1), including XbaI (A/G) and PvuII (T/C) polymorphisms, have been linked to changed receptor activity. These alterations could produce a hormonal environment that supports oncogenesis and HPV persistence [158].

### 2.19. Inflammation Factors in CC Related to HPV

During initial infection, HPV avoids the natural immune reaction, as it replicates without producing notable cytopathic effects or inducing a strong inflammatory response. The virus reduces the release of pathogen-associated molecular patterns (PAMPs) and danger-associated molecular patterns (DAMPs), thus postponing the activation of pattern recognition receptors (PRRs), including Toll-like receptors (TLRs). Chronic HPV infection triggers a sustained inflammatory response that significantly influences the cervical tumor microenvironment. The virus alters cytokine production, disturbs natural immune signaling pathways, affects important transcription factors, and increases the expression of protumorigenic mediators.

The activation of PRRs eventually leads to the stimulation of the transcription of genes involved in inflammation (e.g., TNF-α, IL-6, COX-2, ICAM), proliferation (CDK2), cell survival (XIAP, cIAP, BCL2, BCL-xL), and angiogenesis, promoting the recruitment of innate and adaptive immune cells to the site of infection [132]. Ideally, an effective inflammatory response facilitates viral clearance. Indeed, most HPV infections are transient, with approximately 90% resolving within two years [159].

Chronic inflammation and pro-inflammatory cytokines such as IL-1, IL-6, IL-8, IL-18, COX-2, and TNF-α are significantly involved in CC progression. Cytokines like TNF-α and IL-1α, while inhibitory to normal cervical epithelial cells, stimulate proliferation in HPV-immortalized and cervical carcinoma cell lines through an EGFR-dependent pathway. Chronic inflammation may also disrupt cellular senescence, a critical tumor-suppressive mechanism. Senescent cells release cytokines such as IL-6, IL-18, and IL-1, fostering tumorigenesis. HPV-16/18 E6 proteins promote fibroblast senescence through IL-6/STAT3 signaling, thereby altering the TME and supporting the progression from cervical intraepithelial neoplasia (CIN) to invasive cancer.

Immunosuppressive cytokines like IL-10 are overexpressed in CC and premalignant cervical lesions. HPV E2 protein can bind to the IL-10 promoter, enhancing IL-10 expression and contributing to viral persistence and immune evasion. A shift towards Th2-dominant cytokine profiles, characterized by elevated IL-10 and IL-6 and reduced IFN-γ and TNF-α levels, has been associated with poor immune responses and the progression of cervical lesions. Studies have confirmed decreased IFN-γ and increased IL-10 expression correlating with the severity of CIN lesions. Thus, an imbalance between Th1 and Th2 cytokines facilitates persistent HPV infection and drives lesion progression toward cervical cancer [132,141,160].

The aberrant overexpression of Cyclooxygenase-2 (COX-2) has been implicated in the pathogenesis of various malignancies and inflammatory diseases. Emerging evidence indicates that NF-κB is a key regulator of COX-2 gene expression, with the inhibition of NF-κB activation resulting in the downregulation of COX-2 expression. In the context of HPV-associated carcinogenesis, the viral oncoproteins E6 and E7 have been shown to upregulate COX-2 expression, thereby contributing to both tumor initiation and progression.

A study conducted by Kim et al. specifically examined the impact of the HPV16 E5 oncoprotein on COX-2 expression. Their findings demonstrated that E5 enhances COX-2 expression via the activation of the epidermal growth factor receptor (EGFR) signaling cascade. This upregulation was shown to be mediated, at least in part, through the involvement of NF-κB and AP-1 transcription factors. These results suggest that the E5 oncoprotein may contribute to cervical carcinogenesis by promoting COX-2 overexpression. Table 1 provides an overview of the HPV-encoded proteins implicated in inflammatory processes and cervical cancer pathogenesis [82,132].

### 2.20. MicroRNA Regulation by HPV Oncoproteins: Development of Cervical Cancer

Significant insights have been gained over the last thirty years into the role of genetic aberrations, particularly mutations in protein-coding oncogenes and tumor suppressors, which substantially contribute to the onset of CC. The discovery of non-coding RNAs (ncRNAs), particularly microRNAs as critical drivers of cancer, has transformed the traditional perspective of cancer biology.

These small, non-coding RNA molecules play an essential role in the initiation, progression, and prognosis of CC by regulating several cellular processes, including apoptosis, cell cycle progression, metastasis, and treatment resistance.

MicroRNAs (miRNAs), short non-coding RNAs of around 22 nucleotides in length, facilitate the post-transcriptional regulation of gene expression. They primarily function by engaging with the 3′ untranslated regions (UTRs) of target mRNAs, leading to mRNA degradation or translational repression. This technique enables miRNAs to simultaneously influence the expression of several genes, hence regulating networks of genes involved in diverse cellular activities [161,162,163].

Although the roles of oncoproteins E5, E6, and E7 have been well characterized, they contribute to the complexity of the molecular pathways involved in cervical carcinogenesis through their effects on the regulation of miRNAs [164].

HPV16-E5 expression in keratinocytes has been shown to downregulate miR-203 and miR-324-5p while upregulating miR-146a, further complicating the immune evasion process and facilitating viral persistence [164,165]. The production of E6 and E7 has been demonstrated to activate transcription factors like c-Myc and E2F, which can transactivate miRNA expression, hence promoting tumorigenic alterations [92,166].

A prominent instance of miRNA regulation by HPV oncoproteins is the modulation of miR-23b, which is directly affected by the E6 oncoprotein. The E6-mediated downregulation of miR-23b has been linked to the upregulation of the urokinase-type plasminogen activator (uPA), a protein involved in cancer cell migration and metastasis. The miR-23b and uPA correlation highlights the role of HPV-mediated miRNA modulation in driving cancer cells toward high invasive potential. Furthermore, miR-23b downregulation has also shown an association with radioresistance in cancer cells, indicating that miR-23b may play an important role in therapeutic resistance [164,167].

The other miRNA that has been implicated in HPV-related carcinogenesis is miR-34a, a direct transcriptional target of p53 [168]. E6 is accountable for the inactivation of p53; hence, it is anticipated that the expression of miR-34a is often downregulated in CC. MiR-34a targets many cell cycle regulators, such as CDK4, cyclin E2, and E2F1, in addition to pro-apoptotic and metastatic proteins including Bcl-2 and MET [169,170].

miR-203 is another miRNA that is upregulated or downregulated by HPV oncoproteins. This miRNA is known for its role in driving keratinocytes out of a proliferative state into a terminally differentiated, post-mitotic state. MiR-203 targets the transcription factor p63, which maintains a balance between cellular proliferation and differentiation [168,171,172]. The overexpression of E7 by HPV in differentiated epithelial cells results in the inevitable downregulation of miR-203 expression, which is linked to the preservation of a tumorigenic stem-like phenotype. Additionally, miR-203 was reported to play a key role in regulating the stemness and radioresistance of cancer cells. It has been found that the downregulation of the “stemness” potential of cancer cells is suppressed by miR-203 in several preparations [173,174].

E7 protein has been shown to downregulate miR-203, and thus has been proposed to contribute to the maintenance of stem-like phenotypes, which is often correlated with poor prognosis and treatment resistance. Tumor stem cells, capable of self-renewal and tumor repopulation, are more resistant to radiogenic damage, which adds another layer of complexity in treatment outcomes [168,171].

miR-145 is found to be suppressed in HPV-positive cervical tumors, leading to enhanced oncogene expression and tumor progression [99].

Certain oncogenic miRNAs, such as miR-21, are upregulated in HPV-associated cancers and target tumor suppressor genes such as PTEN and PDCD4, promoting cell survival and resistance to apoptosis [175]. miR-155 enhances inflammatory signaling and modulates the immune environment to favor the immune escape of HPV-infected cells [175,176,177].

A significant study documented the expression profiles of 96 cancer-associated miRNAs in a cohort of 102 CC tumor tissues utilizing quantitative RT-PCR. Among the chosen miRNAs, miR-200a and miR-9 had a strong correlation with overall survival [171,178]. These miRNAs were transfected into HeLa for functional evaluation. The research demonstrated that miR-200a regulates cancer cell migration and metastasis, potentially influencing the metastatic capability of CC cells by modulating pathways associated with cell motility [179,180]. In comparison, miR-9 let us discover regulation pathways for metabolism; therefore, we can understand its role in the metabolism balance of rapidly growing cells like cancer cells [164,178].

These miRNAs actively participate in the start, spread, and maintenance of cervical cancer. Knowing the interaction between HPV oncoproteins and host miRNAs helps to better understand the molecular processes of cervical tumorigenesis and points to new biomarkers and therapeutic targets.

### 2.21. Exosomes, Inflammation, and Microenvironment

Exosomes, nano-sized extracellular vesicles, serve as crucial mediators of intercellular communication within this milieu. They can transport proteins, lipids, mRNA, and microRNA (miRNAs), facilitate horizontal gene transfer, mediate cross-talk between tumor cells and immune cells and other elements of the tumor microenvironment [129,132,181,182,183], generating diverse biological processes, such as tumor progression, immune response, or inflammation [184]. For instance, in HPV-induced CC, exosomes are hypothesized to modulate the balance of pro-inflammatory and anti-inflammatory signals, leading to an environment that promotes immune tolerance and eventual tumor growth [107,185].

Various macromolecules present in exosomes secreted by different CC cell lines, including HeLa cells, have been shown to modulate responses by the immune system. In fact, these exosomes contain anti-apoptotic proteins, like survivin, that are involved in facilitating tumor growth and survival through the prevention of apoptosis. This finding was first published in 2009 and indicated that one of the oncogenic consequences of HPV was the synthesis and release of these extracellular vesicles. In particular, survivin-positive exosomes exerted pro-proliferative properties and had the ability to induce angiogenesis, a critical mechanism implicated in the progression of tumors [132,184,186,187].

Inflammation has an important function in promoting cancer. It is well known that, while the pro-inflammatory cytokines released by immune cells can perpetuate chronic inflammation, chronic inflammation can also perpetuate infection, a process that can be influenced by HPV. For instance, interleukin-36 gamma (IL-36γ), a pro-inflammatory cytokine, has been proposed as a modulator of keratinocyte responses in HPV-induced tissues [132,188,189]. This cytokine triggers signaling pathways, which may play a role in sustaining inflammation, a hallmark of malignant lesions. Moreover, there is evidence that HPV-16 suppresses the expression of certain pro-inflammatory genes, which might indicate that the virus uses mechanisms to avoid immune recognition while promoting inflammation [190].

Exosomes released by HPV-infected cells were shown to transfer inflammatory mediators and mRNAs to neighboring non-infected cells, as described in several recent studies. For example, oncogenic miRNAs like miR-21 and miR-155, which are often upregulated in cancer, are also found in exosomes from HPV-infected cells. The overexpression of these miRNAs has demonstrated anti-apoptotic and pro-proliferative effects, implicating them in immune evasion and tumor survival [132,191,192,193,194]. Another layer of regulatory complexity is added by long non-coding RNAs (lncRNAs), which have been found in exosomes, such as lincRNA-p21 and others, and can modulate gene transcription in accepting cells, potentially promoting inflammation and possibly facilitating tumor growth [191,195,196].

Exosomes stimulate several immune cells, including macrophages, by activating pro-inflammatory signaling pathways. Exosomes derived from cancer cells can stimulate the macrophage NF-κB pathway, resulting in the release of pro-inflammatory cytokines such as TNF-α, IL-6, and CCL2 [130,131,132]. These cytokines then stimulate immune cell recruitment to the tumor site, resulting in a host environment that facilitates tumor progression. Remarkably, exosomes from keratinocytes harboring HPV can additionally transport IL-36γ, a pro-inflammatory cytokine that activates Wnt signaling pathway-dependent pro-inflammatory responses in neighboring keratinocytes. These findings indicate the significant role of exosomes in maintaining an inflammatory cycle within the cervical epithelium that could enhance HPV persistence and tumorigenesis [132,188,189].

The immune landscape in CC is even more complex considering that exosomes also interact with numerous immune cells, including dendritic cells and T cells. Exosomes can deliver viral antigens and cytokines that modify immune cell activation and polarization. The expansion of regulatory T cells or the inhibition of cytotoxic T cell activity can result from this interaction, allowing the tumor to evade immune surveillance [109].

Finally, there is an interest in the therapeutic potency of inhibiting inflammation in the HPV-related CC context. We believe that strategies to either inhibit the generation of exosomes or neutralize their pro-inflammatory effects hold promise for new therapies. HPV is recognized as an infectious agent that induces chronic inflammation, and the involvement of inflammation in cervical carcinogenesis has provided new insights into prevention and treatment through its processes.

### 2.22. Epigenetic Modifications in CC

Not all HPV infections develop into cancer, suggesting that other factors control carcinogenesis. Among these, epigenetic changes, such as DNA methylation, are most important in controlling host and viral gene expression during HPV infection. Epigenetics is associated with modifications in gene function without changes in the DNA sequence. These changes consist of non-coding RNA-mediated control, histone modification, and DNA methylation.

Epigenetic DNA methylation occurs at the C5 site of cytosine residues inside CpG dinucleotides and is linked to the recruitment of transcriptional repressors or the suppression of transcription factor DNA-binding activity [90,197,198,199]. By changing the expression of important regulating genes, aberrant methylation patterns, including global hypomethylation and the promoter hypermethylation of tumor suppressor genes, help to drive malignant transformation in cancer [200].

Although the exact link between hr-HPV and cellular DNA methylation is still being explored, mounting evidence suggests that hr-HPV may be involved in the mis-regulation of the methylation-associated patterns of miRNA, subsequently resulting in the dysregulation of tumor-suppressive or oncogenic miRNAs [90,201].

The methylation of adenomatous polyposis coli (APC), an important negative regulator of β-catenin, leads to the hypermethylation of the APC promoter in HPV-infected CC cells, promoting β-catenin accumulation in the cell. These findings indicate that APC hypermethylation occurs at early phases in HPV carcinogenesis, but in advanced disease, APC hypermethylation is also intensified and underscores the importance of epigenetic mutations in tumor progression [107,202].

Frequently hypermethylated in CC are tumor suppressor genes, such as KIP1 (p27), TP53 (p53), PTEN, and RASSF1, all of which influence cell cycle control, proliferation, and death. For example, PTEN methylation stimulates PI3K signaling pathway activation, thus promoting cellular proliferation [198,201,203].

Other common epigenetic alterations include the silencing of the CDKN2A gene, which encodes the tumor suppressor protein p16^INK4a^. The hypermethylation of CDKN2A leads to pRB phosphorylation and results in releasing E2F and aberrant cell cycle progression, contributing to cervical carcinogenesis [201,204,205].

In HPV-induced cervical carcinogenesis, the hypermethylation of the CDH1 promoter is linked to cell adhesion, promoting invasion and metastases and influencing tumor progression. Levels of CDH1 methylation are positively associated with the severity of CC and have therefore been suggested as an epigenetic biomarker for the determination of CC severity and prognosis [201,206].

Oncogene hypomethylation has also been shown to be associated with CC progression, in contrast to the hypermethylation of tumor suppressor genes. In cancer, STK31 expression is found to be upregulated by promoter hypomethylation, which in turn is a predicted driver of cancer cell migration and invasion. The STK31 promoter exhibits substantial methylation in HPV-negative tumors, whereas it undergoes demethylation in high-risk HPV infections, leading to a marked overexpression in CC cell lines triggered by either HPV16 or HPV18 [207].

Invasive cervical carcinoma exhibits an elevated incidence of LCR methylation, particularly in HPV16-positive tumors [208,209]. Furthermore, E2 is modulated by hypermethylation at the E2 binding site (E2BS) inside the LCR, resulting in the overexpression of the E6 and E7 oncogenes, akin to the phenomenon found following the integration of the viral genome [208]. LCR methylation density analysis indicates the subtype-specific epigenetic risk of high-risk HPVs, where HPV16 appears to have the highest level of methylation density compared to other high-risk types like HPV18 and HPV45.

The most promising epigenetic markers include the hypermethylation of hsa-miR-124-2, SOX1, TERT, and LMX1A, which have emerged as potential biomarkers for CC precursors, regardless of high-risk HPV status. Rogeri et al. reported a correlation between hsa-miR-124-2 hypermethylation and the reduced expression of miR-124, and proposed its involvement in cervical carcinogenesis. Beyond miR-124, Yao et al. identified miR-432, miR-1286, miR-641, miR-1290, miR-1287, and miR-95, which were correlated with hr-HPV genetic status, as a panel of hypermethylated miRNA for CC. Wilting et al. also showed that increased promoter methylation resulted in the transcriptional repression of hsa-miR-149, hsa-miR-203, hsa-miR-375, and hsa-miR-638 in CC. Yao et al. further showed that the downregulation of hsa-miR-432, hsa-miR-1286, hsa-miR-641, hsa-miR-1290, hsa-miR-128, and hsa-miR-95 in primary cervical tumors is dependent on their methylation status [175,176,177].

Wilting et al. reported significantly elevated levels of methylation of hsa-miR-203, hsa-miR-149, and hsa-miR-375 in patients with CC, with hsa-miR-203 and hsa-miR-375 showing higher levels in high-grade lesions. Overall, these discoveries highlight the importance of miRNA methylation in cervical carcinogenesis. The methylation of tumor-suppressive miRNAs, such as hsa-miR-124, hsa-miR-203, hsa-miR-375, and hsa-miR-149, is particularly clear and shows aberration in CC progression through the downregulation of these important regulatory pathways [176,177,201].

CIN progression is also associated with methylation changes; hence, methylation markers can be useful for the stratification of lesions and prognostication. Vink et al. showed that when lesions evolved from CIN2 to CIN3, both p16 and Ki-67 expression increased, while E4 expression decreased along with increasing miR-124-2 methylation, indicating their importance in determining disease severity [197,210].

Cervical carcinogenesis caused by HPV could depend on the dysregulation of methylation in both viral and host genomes. Knowing about these epigenetic changes opens doors for early diagnosis, prognosis, and treatment plans in HPV-related tumors.

## 3. Prevention and Management

### Vaccination

Vaccination against HPV has the potential to prevent CC, as well as other disorders that are related with the virus [211]. In 2018, the World Health Organization advocated for global efforts to eradicate CC, establishing a benchmark of four cases per 100,000 women annually [212]. The most effective method of preventing HPV infection is the vaccine, which has been available to medical professionals in the US since 2006 and is now the standard of treatment [213,214].

Recent studies support the extension of the HPV vaccination from a strictly preventive action to a possible therapeutic adjuvant for individuals already infected. Particularly for the genotypes covered by the 9-valent HPV vaccination, the administration of the vaccination in a cohort of HPV-positive women was linked to a noticeably improved clearance rate of the infection. In vaccinated women, clearance rates reached 72.4%; in the unvaccinated cohort, they were 45.7%; and those who had also had surgical removal of high-grade lesions showed the most notable benefits. These data challenge the accepted limit of HPV vaccination to pre-exposure application and show a synergistic benefit when given post treatment, presenting a promising secondary prevention method for patients susceptible to reinfection or lesion recurrence [215].

Especially against high-risk carcinogenic types like HPV-16 and HPV-18, the HPV vaccination has shown notable subtype-specific efficacy. Data from 22 randomized controlled studies comprising more than 95,000 participants revealed a 46% relative decrease in CIN2+ lesions linked to HPV-16/18 and a 73% reduction in CIN3+ lesions, thus underlining the critical importance of the vaccination in the prevention of pre-cancerous cervical abnormalities. The immunological durability of the current vaccination formulations is highlighted by the long-term follow-up data showing continuous protection for over 12 years. These results support the case for broad immunization policy within comprehensive cervical cancer prevention campaigns and confirm the need for early vaccination before HPV exposure [216].

Although the Centers for Disease Control and Prevention has reaffirmed its earlier advice that children between the ages of 11 and 12 should receive the HPV vaccine, only 58.6% of adolescents followed the immunization schedule in the year 2020 [217,218,219].

Although there are now efficient HPV vaccines, reaching enough population-level immunity calls for focused campaigns to increase vaccination acceptance, especially among teenagers. Coverage has been raised with success by school-based immunization programs, community education, and provider advocacy. Attaching herd immunity to several HPV subtypes—especially those not covered by earlier vaccine generations—requires increasing vaccination rates. These treatments help to prevent main infections and lower the frequency of high-risk HPV strains by extending coverage and addressing sociocultural barriers, possibly benefiting persons already exposed by lowering their chances of reinfection or chronic infection [220].

## 4. Future Directions

### 4.1. Research Gaps

The adoption of preventative techniques is severely hampered by the low level of information that is available among individuals who are considering careers in the healthcare industry. It is vital to adopt a comprehensive array of measures to meet the World Health Organization’s goal of eradicating CC by the year 2030. These measures include the design of policies, education for high-risk groups, and the provision of training and information for healthcare professionals.

Since its introduction, both supporters and opponents of HPV vaccination have expressed their views [219]. The assertions made by anti-vaccination organizations on social media about the possible adverse effects of vaccines are unsupported by any data [221,222].

Negative coverage of HPV vaccination can cause parents to feel confused and anxious, which can lead to healthcare practitioners assuming that parents do not value the vaccine and create an awkward conversational environment. Understanding successful social media tactics is essential for promoting HPV vaccination and battling bad PR. This entails determining which unfavorable plotlines require attention, as well as when, how, and by whom they should be addressed.

Important gaps are those that deal with systemic strategies (for example, best practices for health insurers and plans), healthcare provider initiatives (for example, getting providers involved in quality improvement efforts in-clinic], or social media and vaccine confidence [for example, using social media to boost vaccine confidence). It is very important to create and test treatments in all these areas in order to fill in the gaps and find the best ways to increase the number of people who get vaccinated against HPV.

The quadrivalent preventive HPV vaccine has shown a 70% decrease in the occurrence of cervical and other HPV-associated cancers in young men and women, with a single dose offering protection for at least ten years [223].

The significance of early delivery is evidenced by the observation that anti-HPV antibody titers were 2–3 times greater in younger persons post immunization [224].

There are significant knowledge gaps among medical professionals on HPV infection, vaccination, and preventative strategies, according to several studies that were conducted in high-income countries [HICs], as well as low-income countries and middle-income countries. To achieve the long-term aims of lowering the number of cancers that are caused by HPV and the WHO’s vision of reaching the goal of eliminating CC by the year 2030, it is necessary to address these discrepancies. The success of immunization efforts will increase because of this, and will also make it possible to accomplish long-term objectives [225,226,227].

First things first, if we want to understand how medical schools and curricula influence the dissemination of accurate information on HPV prevention techniques, we need to evaluate the degree of knowledge that medical students and residents gain. It is imperative that this precaution be taken because of the rising incidence of non-CCs that are caused by HPV, the unwillingness to receive vaccines, and the poor participation rates in CC screening programs.

The analysis reveals major barriers to getting tested for CC, even though CC can be prevented in most cases by appropriate screening and immunization against HPV. Some of the most common concerns include the anticipated discomfort associated with the testing procedure (which was reported by as many as 63% of respondents in certain countries) and the discomfort of disclosing sexual history to healthcare professionals [which was reported by as many as 57% in various communities). These difficulties are further exacerbated in countries with poor incomes, which are locations where ninety percent of deaths from CC occur [14].

### 4.2. Public Health Implications

Implementing effective control and prevention measures, such as an HPV vaccination and improved screening, should bring about a reduction in the number of malignancies that are caused by HPV. Researchers make use of information obtained from the National Cancer Registry Program (NCRP) in order to assess the degree to which HPV-related measures have improved both outcomes and prognoses.

Healthcare institutions all over India have started to demand screenings for common disorders, such as oral and cervical cancers, as part of their attempts to limit the number of people who are diagnosed with cancer. The objective of this screening program is to bring the cancer rate down to a lower level [228].

Cancer registries maintained in hospitals and communities across India serve as vital sources for assessing the impact of primary (e.g., vaccination) and secondary (e.g., screening) prevention programs. A comprehensive understanding of the epidemiology and transmission dynamics of HPV-related cancers is important to guide preventive measures. Such insights are critical for nationwide efforts aimed at reducing the incidence of these cancers before they occur, ultimately improving public health outcomes. The purpose of this study is to investigate the spread of HPV-related cancers by evaluating data from India’s National Cancer Registry Program, Hospital-Based Cancer Registries (HBCRs), and Population-Based Cancer Registries (PBCRs) [229,230].

In light of the fact that precancerous lesions seldom manifest themselves, it is essential to undergo CC screening on an annual basis, even after receiving an HPV vaccine. A high-performance HPV test and at least two other tests should be administered to every individual before they reach the ages of 35 and 45, respectively, according to international policymakers who have mandated that this be carried out. At the age of thirty, women should begin having checks for CC every five to ten years. This screening should be performed more frequently. It is recommended that women who are HIV positive begin getting tested every three years once they reach the age of 25.

Women may want to collect their own HPV testing samples, because they have been confirmed to be just as reliable as those collected by medical professionals.

The early detection and treatment of CC could lead to a cure. Initially, one can identify the indicators through knowledge, and thereafter, if practicable, pursue medical aid to address any arising difficulties [14].

Usually, formal referral to treatment providers follows the confirmation of a diagnosis. The diagnosis process depends critically on clinical tests and investigations. The prevalence of HPV16/18 and the potential for cross-protection underscore the necessity of incorporating more affordable immunizations in underdeveloped countries.

According to the World Health Organization’s Global Strategy, which sets three targets for 2030, the goal is to reduce the annual incidence of new cases to four or fewer per 100,000 women. This approach directs all nations toward the eradication of the disease in the next decades:

- By the age of 15, 90% of females will have been administered the HPV vaccine;
- By the ages of 35 and 45, 70% of women will have received a high-quality screening;
- Ninety percent of women with cervical disease will receive treatment [14].

If this elimination goal is achieved, modeling indicates that 74 million new CC cases and 62 million fatalities could be prevented by 2120. Analysis of cervical cancer (CC) data compiled by the World Health Organization (WHO) and other international stakeholders highlights a collective global effort aimed at the elimination of cervical cancer and its related health burdens [14].

Identifying and inhibiting carcinogenic HPV genotypes has emerged as a crucial and efficient objective for CC prevention and care, thanks to the substantial decrease in the worldwide incidence of CC caused by HPV vaccines [9,16,231]. Pap smears and other HPV detection methods have been incorporated into standard cervical screening programs in industrialized countries, which has resulted in the complete elimination of invasive CC in these countries. Since the previous staining method was shown to be imprecise, it is reasonable to consider switching to liquid-based cytology [232,233].

The prophylactic HPV vaccine may serve as a cost-effective public health intervention in low- and middle-income countries, which are generally marked by inadequate quality screening programs [234,235]. Historically, the field’s reliance on Pap tests has slowed the implementation of this technology [236].

Because of inequalities in providing education, laboratory resources, and financing, primary HPV testing is not easily accessible to most people. One of the non-emergency treatments that the COVID-19 outbreak impacted is CC screening [237]. There are substantial obstacles to healthcare services, including HPV testing, for women living in low-income neighborhoods.

There have been certain countries in western Europe and sub-Saharan Africa that have started adopting self-sampling at home as an innovative alternative of the screening that takes place in medical facilities [238]. Women in the United States who do not have easy access to doctors or who are scared to obtain the requisite internal exam for screening would benefit tremendously from the broad adoption of this strategy [239].

In order to strike a balance between the inherent advantages of accessibility and the potential drawbacks of incorrect handling and sample collection, regulatory considerations and implementation plans would be required. The provision of instructions that are straightforward, based on images, and at a level of literacy that is functional will improve the conduciveness of the environment, particularly among the most vulnerable groups of women [14,230].

## 5. Conclusions

HPV stands as the primary viral cause of cervical cancer [CC], which maintains its status as a worldwide public health concern. Extensive research has been conducted to understand HPV-induced carcinogenesis at the molecular level, with particular attention given to the E6 and E7 oncogenes that drive tumorigenesis. Targeted therapeutic approaches against these oncoproteins have demonstrated promising results. However, recent research findings revealing the E5 oncogene’s role in cervical cancer progression indicate the need for broader molecular targeting in therapeutic development.

HPV oncoproteins, along with immune evasion mechanisms and epigenetic modifications, work together to create the complex process of cervical carcinogenesis. The transformation of cervical epithelial cells involves two primary molecular events, including alterations in miRNA expression and DNA methylation. Notably, the hypermethylation of tumor-suppressor miRNA promoters, resulting in the dysregulation of critical cellular pathways, has emerged as a central factor in the advancement of HPV-induced malignancies. Gene expression regulation in CC becomes more complex due to HPV’s ability to modify miRNA expression at critical genomic locations, which requires the development of new therapeutic approaches to address these epigenetic and molecular disruptions.

Research has successfully uncovered HPV-related CC molecular mechanisms, but the clinical application of these findings continues to be difficult. Today’s HPV treatments, including vaccinations and epigenetic inhibitors, encounter multiple barriers in their implementation, including accessibility issues, along with specificity limitations and possible unwanted side effects. Advanced delivery systems combined with epigenetic therapies show great potential for boosting therapeutic outcomes. Future research must focus on refining these approaches to ensure that they are both cost-effective and capable of reducing the global burden of CC.

The prevention of cervical cancer requires an approach that combines multiple protection layers. A preventive strategy must begin with HPV vaccination as the primary intervention, followed by the effective monitoring and treatment of precancerous lesions as the secondary prevention, and then the timely diagnosis and treatment of invasive cancer as the tertiary prevention. All individuals between 9 and 16 years of age must receive the HPV vaccine according to national immunization schedules, to prevent infection-related malignancies.

Medical education should incorporate comprehensive information on HPV, including its role in non-cervical cancers, to raise awareness, advocate for vaccination, and support healthcare professionals with the necessary knowledge to sustain HPV prevention efforts.

In conclusion, the reduction in CC incidence worldwide depends on improved global HPV vaccination programs and ongoing molecular and epigenetic research advancements.

## Figures and Tables

**Table 1:** Influence of HPV oncoproteins on immune system components.

Immune Component	HPV Strategy	Involved Viral Proteins
Th1/Th2 balance	Shift to Th2 phenotype (immunosuppression)	E6, E7
CD8⁺ CTLs	Impaired antigen presentation via MHC-I downregulation	E5, E7
Tregs	Expansion and increased IL-10/TGF-β production	E7
TAMs	Recruitment and pro-tumor cytokine secretion	E5, E6
